# Post-revascularization Ejection Fraction Prediction for Patients Undergoing Percutaneous Coronary Intervention Based on Myocardial Perfusion SPECT Imaging Radiomics: a Preliminary Machine Learning Study

**DOI:** 10.1007/s10278-023-00820-1

**Published:** 2023-04-14

**Authors:** Mobin Mohebi, Mehdi Amini, Mohammad Javad Alemzadeh-Ansari, Azin Alizadehasl, Ahmad Bitarafan Rajabi, Isaac Shiri, Habib Zaidi, Mahdi Orooji

**Affiliations:** 1 Department of Biomedical Engineering, Tarbiat Modares University, Tehran, Iran; 2 Division of Nuclear Medicine and Molecular Imaging, Geneva University Hospital, CH-1211 Geneva 4, Switzerland; 3 Cardiovascular Medical and Research Center, Iran University of Medical Sciences, Tehran, Iran; 4 Echocardiography Research Center, Rajaie Cardiovascular Medical and Research Center, Iran University of Medical Sciences, Tehran, Iran; 5 Cardio-Oncology Research Center, Rajaie Cardiovascular Medical and Research Center, Iran University of Medical Sciences, Tehran, Iran; 6 Geneva University Neuro Center, Geneva University, Geneva, Switzerland; 7 Department of Nuclear Medicine and Molecular Imaging, University of Groningen, University Medical Center Groningen, Groningen, Netherlands; 8 Department of Nuclear Medicine, University of Southern Denmark, Odense, Denmark; 9 Department of Electrical and Computer Engineering, University of California–Davis, Davis, CA USA

**Keywords:** Ejection fraction, Machine learning, Myocardial perfusion imaging, PCI, Quantitative features, Radiomics

## Abstract

In this study, the ability of radiomics features extracted from myocardial perfusion imaging with SPECT (MPI-SPECT) was investigated for the prediction of ejection fraction (EF) post-percutaneous coronary intervention (PCI) treatment. A total of 52 patients who had undergone pre-PCI MPI-SPECT were enrolled in this study. After normalization of the images, features were extracted from the left ventricle, initially automatically segmented by k-means and active contour methods, and finally edited and approved by an expert radiologist. More than 1700 2D and 3D radiomics features were extracted from each patient’s scan. A cross-combination of three feature selections and seven classifier methods was implemented. Three classes of no or dis-improvement (class 1), improved EF from 0 to 5% (class 2), and improved EF over 5% (class 3) were predicted by using tenfold cross-validation. Lastly, the models were evaluated based on accuracy, AUC, sensitivity, specificity, precision, and F-score. Neighborhood component analysis (NCA) selected the most predictive feature signatures, including Gabor, first-order, and NGTDM features. Among the classifiers, the best performance was achieved by the fine KNN classifier, which yielded mean accuracy, AUC, sensitivity, specificity, precision, and F-score of 0.84, 0.83, 0.75, 0.87, 0.78, and 0.76, respectively, in 100 iterations of classification, within the 52 patients with 10-fold cross-validation. The MPI-SPECT-based radiomic features are well suited for predicting post-revascularization EF and therefore provide a helpful approach for deciding on the most appropriate treatment.

**Supplementary Information:**

The online version contains supplementary material available at 10.1007/s10278-023-00820-1.

## Introduction

Cardiovascular diseases are the most threatening diseases globally and cause mortality [1]. Coronary artery disease (CAD) is one of the primary causes of cardiovascular diseases [2]. About half of the heart failure cases have left ventricular dysfunction, as indicated by estimates of 650,000 new heart failure cases annually. For more than two-thirds of the patients with left ventricle (LV) dysfunction, CAD is the leading cause [3]. Myocardial infarction (MI) leads to LV systolic dysfunction, correspondingly LV dilatation, and eventually heart failure, leading to decreased quality of life [4]. Percutaneous coronary intervention (PCI), coronary artery bypass graft (CABG) surgery, and medical therapy are the main recommended treatment plans for CADs [5, 6]. Revascularization improves the viable myocardium function [7], but it is not feasible or beneficial for all patients [8]. Aside from being costlier than PCI, CABG requires longer hospitalization and has more complications. Nevertheless, an effective method is needed to predict the outcome of revascularization before PCI. An important parameter for evaluating cardiac function is the ejection fraction (EF), which is the amount of blood ejected from the heart. EF, as an indication of LV systolic efficiency, is determined by the calculation of LV end-diastolic and end-systolic volumes. Even though it usually refers to the LV, it can also be a biomarker of the pumping ability of the heart as well as types of heart failure.

Stenosis of coronary arteries is being diagnosed by coronary angiography (CAG), which is the gold standard approach [9]. However, CAG is costly and invasive, and it comes with complications such as infection and causing damage to the catheterized artery, thus, quests alternative approaches. The myocardial perfusion imaging with single-photon emission computed tomography (MPI-SPECT) test is often considered one of the most accurate and essential non-invasive cardiac imaging tests. The MPI-SPECT provides crucial diagnosing information for a wide variety of cardiovascular diseases and helps assess treatment effectiveness. MPI-SPECT is primarily used to diagnose CAD, stratify patients based on their risk for CAD, assess therapy and myocardial viability, and guide patients through a PCI or CABG [10]. MPI-SPECT has been the most regularly employed non-invasive imaging technique for assessing CAD at low or intermediate risk [11]. Pharmacological stress testing must be considered in cases where exercise stress testing is contraindicated. The most commonly used radiopharmaceuticals are Thallium-201 (^201^Tl chloride), Technetium-99m sestamibi, and Technetium-99m tetrofosmin. In order to assess viability, Thallium-201 is favored, but is not preferred to evaluate the LV. Technetium-99m sestamibi is preferred for evaluating LV function compared to Thallium-201 [12–14].

Radiomics is an emerging field in which extracting different features from digital images is used for prediction, diagnosis, and prognosis via machine learning approaches [12, 15–20]. The role of radiomics and machine learning in cardiology has been demonstrated in several studies via different imaging modalities. Arsanjani et al. [21] employed a model-based approach to determine whether early revascularization can be effectively predicted using clinical data and quantitative features derived from perfusion SPECT imaging. The model was trained on various clinical and imaging variables, including patient demographics, clinical history, and SPECT image features. The primary endpoint was the need for revascularization. The machine learning model achieved an area under the ROC curve (AUC) of 0.81 for the prediction of revascularization. The most important predictors of revascularization were combined supine/prone total perfusion deficit (TPD) and supine stress TPD. However, there were some limitations in this study. The MPS protocol used was dual-isotope imaging, which is limited by difficulties in comparing rest and stress images due to differences in image resolution and patient radiation exposure. Moreover, the machine learning model was based on global perfusion abnormalities rather than regional abnormalities. Wang et al. [22] utilized LV tomograms obtained from D-SPECT-MPI for auxiliary diagnosis to assess radiomics methods' feasibility and effectiveness. The predictive models had a sensitivity within [86–91%] and a specificity within [91–95%]. These results suggest that radiomics has the potential as a useful tool for the auxiliary diagnosis of myocardial ischemia in patients with CAD. Nevertheless, it suffers from some limitations. One of the limitations was delineating lesions manually and defining the edges of ischemic areas, which had obvious boundaries that caused under and over-estimation. Ashrafinia et al. [23] investigated the prediction of coronary artery calcification using MPI-SPECT radiomic features. They have also indicated a significant correlation between perfusion heterogeneity and coronary artery calcification scores. While the study did find that radiomics analysis can help identify the presence of significant coronary artery stenosis, it did not investigate whether this information can improve patient outcomes. Based on non-contrast Cine cardiac magnetic resonance (Cine-CMR) images, Avard et al. [24] developed a machine-learning approach to differentiate MI and viable tissues/normal cases. Their study showed that using radiomics analysis on non-contrast Cine-CMR images makes MI detection more accurate. The best-performing machine learning algorithm achieved an area under the ROC curve of 0.93 (accuracy = 0.86, recall = 0.87, precision = 0.93, and F1-score = 0.90) by logistic regression in multivariate analysis, indicating high accuracy in detecting MI. Furthermore, the study also investigated the individual radiomics features that were most strongly associated with the presence of MI. They found that features related to the myocardium’s intensity, texture, and shape were most strongly associated with MI. However, the study did not provide detailed clinical information about the patients with MI, such as the severity of their condition or their medical history, which could have impacted the radiomics features and the performance of the machine learning algorithms. Most recently, Arian et al. [25] utilized radiomic features extracted from late gadolinium enhancement on cardiac MR (LGE-CMR) images to predict elevations in the myocardial function of patients undergoing CABG and achieved promising results. The model had an AUC of 0.78 and a sensitivity of 82%. Nevertheless, the study did not evaluate the impact of potential confounding factors, such as medication use, comorbidities, and lifestyle factors, on the relationship between radiomic features and changes in myocardial function after CABG. Sabouri et al. [26] highlighted MPI-SPECT radiomics potential in identifying the left ventricular contractile pattern, which was shown to be associated with cardiac resynchronization therapy response. The results of the study showed that the machine learning algorithms were able to accurately classify SPECT images into U-shaped and non-U-shaped left ventricular contractile patterns with high accuracy. The MLP algorithm achieved the highest AUC (80%) and sensitivity (85%) among ConQuaFea (conventional quantitative features, such as phase analysis and QGS features) models, whereas gradient boosting achieved an AUC of 78% and sensitivity of 92% among combined models (radiomics + ConQuaFea). However, the criteria were based on the left ventricular contractile pattern and didn't consider factors, such as the lead location for CRT.

In this study, the main goal was to test whether radiomic features can accurately predict post-PCI EF and differentiate revascularization outcomes and evaluate the accuracy, AUC, sensitivity, specificity, precision, and F-score of the models developed using the radiomic features. This study hypothesizes that radiomic features extracted from MPI-SPECT can accurately predict post-PCI EF and differentiate between patients who will experience no or dis-improvement, those with improved EF of less than 5%, and those with improved EF over 5%. Furthermore, by using machine learning algorithms to aid physicians with medical image interpretation, this computer-aided diagnostic approach may improve the accuracy of diagnosis. Ultimately, we hypothesize that this approach can lead to better treatment decisions and outcomes for patients undergoing revascularization procedures.

## Materials and Methods

**Fig. 1:**
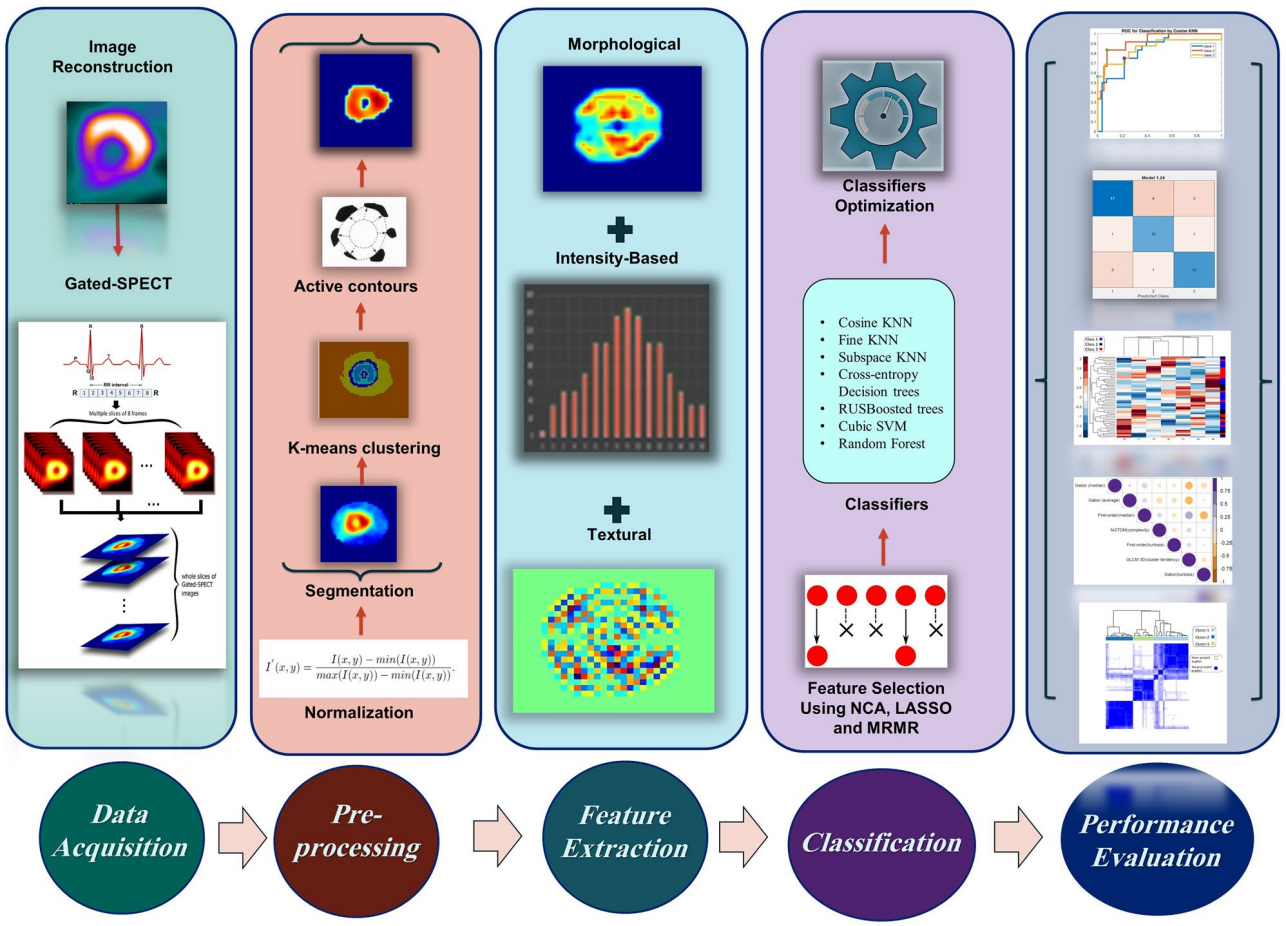
An infographic flowchart summarizing different steps of the study from data retrieving to preprocessing, feature extraction, classification, and finally, performance evaluation of the proposed models

An infographic illustration of the workflow followed in this study is presented in Fig. 1.

### Study Population

Patient’s clinical and imaging data have been collected retrospectively at Rajaie Cardiovascular Medical and Research Center, Tehran, Iran. This study was conducted in accordance with international ethical standards considering the institutional recommendations and the 1964 Helsinki declaration and its later amendments. This retrospective study was approved by the ethics committee of Iran University of Medical Sciences (IR.IUMS.FMD.REC.1400.087). Inclusion criteria included patients who had both prior and post-PCI echocardiographic reports, with an interval of fewer than 6 months between MPI-SPECT, echocardiography, and PCI. In addition, only patients who had undergone a ^99m^Tc-MIBI scan were enrolled in this study, whereas patients who had undergone a ^201^Tl scan were excluded. Moreover, we excluded studies where significant extracardiac activity or motion artifacts caused by patient movement were present. Eventually, 52 patients were enrolled. The characteristics of the patients are presented in Table 1. For categorical data (gender), we used Fisher’s exact test due to the small sample size and the expected frequency count not being obtained in the chi-square test. For continuous data analysis, we employed the Wilcoxon rank-sum test, which is appropriate for non-parametric data. Our results indicate no statistically significant difference between pairs of groups except for one case, specifically the post-EF comparison between class 1 and class 3.

**Table 1:** Characteristics of patients based on three different classes. C1C2, C1C3, C2C3 indicate pairs of class 1 and class 2, pairs of class 1 and class 3, and pairs of class 2 and class 3, respectively

	No or dis-improvement (class 1)	Improved EF from 0 to 5% (class 2)	Improved EF of over 5% (class 3)	*P* values
Number	24	12	16	
Age (years)	59.58 ± 8.93	53.67 ± 11.53	60.38 ± 10.00	C1C2: 0.13 C1C3: 0.6 C2C3: 0.09
Gender (male/female)	18/6	9/3	10/6	0.7256
Ejection fraction (pre-PCI) (Echo) (%)	45.83 ± 11.24	43.75 ± 7.11	39.06 ± 7.75	C1C2: 0.32 C1C3: 0.06 C2C3: 0.17
Ejection fraction (post-PCI) (Echo) (%)	42.71 ± 10.70	48.75 ± 7.11	50.00 ± 6.61	C1C2:0.05 C1C3: 0.02 C2C3: 0.07

### Data acquisition

All registered patients underwent conventional MPI with electrocardiography-gated SPECT (gSPECT) for clinical purposes. A gSPECT scan was conducted after injection of 15–20 mCi of Technetium-99 m sestamibi. Planar SPECT images were acquired using a dual-headed gamma camera (Symbia T2, Siemens Healthcare) installed with automatic body contouring of 135 (RAO) to − 45 (LAO) with a standard resting protocol. The cardiac-gated protocol was used to obtain 32 projections (30 s per projection and 16-bin gating) of 64 by 64 matrix size (0.48 pixels at zoom 1.33) and 180° from right anterior to left anterior oblique. The system was assumed to have a 9.7% energy resolution, and counts were collected between 112 and 168 keV (140 keV–20 keV energy windows). Throughout the image acquisition, the patients were asked to stay in the supine position. Images were reconstructed using filtered back projection (FBP) with a Butterworth post-reconstruction filter (order = 5, cutoff frequency of 0.45 cycles/mm).

### PCI Protocols

The CAG was done for all patients via radial or femoral artery approaches. The PCI (angioplasty with stent) was done for those with significant CAD. A significant CAD was defined as more than 50% narrowing of the diameter of the lumen of the main coronary artery and/or more than 70% diameter narrowing of the lumen of the left anterior descending coronary artery, left circumflex artery, or right coronary artery. As the ground truth of post-revascularization improvement, echocardiography was performed prior to and post-PCI. EF values of echocardiographic reports were considered in three classes; class 1: no increase or decreased EF, class 2: 5% improvement, and class 3: improved EF of over 5% (EF was reported by steps of 5% by the echocardiography). The number of patients per class and mean ± standard deviation of their EF pre- and post-PCI are presented in Table 1.

### Preprocessing and Segmentation

Before feature extraction, the gray-level normalization method was applied to all images. Equation 1 defines gray-level normalization between zero and one.

1 $$I'\left(x,y\right)=\frac{I\left(x,y\right)-min(I\left(x,y\right))}{max(I\mathit{\left({x,y}\right)})-min(I\left(x,y\right))}$$

Automatic segmentation was applied to the axial view of the SPECT images to delineate the left ventricle. At first, the k-means clustering algorithm was applied. Regions of interest (ROIs) and some regions outside ROIs were selected. Secondly, we used Snake active contour model [27] to define the delineations better. Finally, an expert radiologist edited and confirmed the automatic segmentation of the left LV to ensure its validity.

### Feature Extraction

The feature extraction involved extracting 2D and 3D features from short-axis images using two different tools, including in-house-generated codes via MATLAB 2019b (Mathworks, Natick, MA, USA), and standardized environment for radiomics analysis (SERA), which is a MATLAB-based package based on guidelines from the Image Biomarker Standardization Initiative (IBSI) [28, 29]. Figure 1 illustrates an overview of the radiomic analysis pipeline that attempts to predict the effect of revascularization on EF. Using our in-house-generated feature extractor, per-pixel (extracted for each pixel using a kernel around the pixel sliding over the image) and per-image (extracted from the whole image) 2D textural features were extracted from normalized images. These features included three different feature groups, namely, Haralick gray level co-occurrence matrix (GLCM) and LAWS features which are texture-based, and Gabor features which are transform-based.

For per-pixel features, kernels of sizes 3 × 3, 5 × 5, and 7 × 7 were considered for calculating each feature, and then statistical analysis was exerted on extracted values from the pixels of each 2D image. Statistical parameters were average, variances, median, skewness, and kurtosis. For the co-occurrence matrices, three parameters were considered and tuned: distance: 1, orientations: 0, 45, 90, 135, and quantization levels: 4, 8, and 16 [30–32]. LAWS texture features can effectively measure edges, waves, ripples, levels, and spots. It contains three vectors that indicate averaging, edges, and spots. These vectors are convolved with themselves and with each other leading to five vectors: level, edge, spot, ripple, and wave. LAWS mask is a variable parameter through the image. In our study, we used masks of different sizes.

In addition to our in-house generated feature extractor, radiomic features were extracted using the SERA package. It extracted 269 radiomic features, including 50 statistical first-ordered, 29 morphological, and 190 3D textural features (50 GLCM, 32 GLRLM, 32 GLSZM, 32 GLDZM, 10 NGTDM, and 34 NGLDM). All feature values were normalized by z-score. Table 2 represents the extracted features and their descriptions.

**Table 2:** The number of extracted features from different feature extractor tools feature families, types, and groups. The fifth column indicates whether features were extracted from two-dimensional images (per slice) or image volumes. Finally, the last column indicates the per-pixel or per-image feature extraction approach

Origin of code	Feature type	Feature group	Number of features	2D/3D	Per-pixel/per-image
SERA package	Morphology	-	29	3D	Per-image
	Textural	GLCM	50	3D and 2D	
		GLRLM	32		
		GLSZM	32		
		GLDZM	32		
		NGTDM	10		
		NGLDM	34		
	First-order Intensity-based	-	50	-	Per-image
Self-generated	Textural	GLCM/ Haralick	14	2D	Per-pixel
		LAWS	25		Per-pixel
	Transform-based	Gabor	48		Per-image

### Feature Selection

Feature selection methods were applied to reduce the risk of overfitting and avoid the curse of dimensionality. neighborhood component analysis (NCA), minimum redundancy maximum relevance (MRMR), and least absolute shrinkage and selection operator (LASSO) were feature selection methods that were used. Feature selectors were set to select 3 to 10 features, and feature sets of 7 features achieved the best results. In order to compare feature values between the classes, as a non-parametric statistical test, the Wilcoxon rank-sum was used between groups. In addition, the Wilcoxon rank-sum test with *p* value < 0.05 was used to determine if the difference was statistically significant. The abbreviated names of the selected features along with their information, including the type, group, defined parameters (descriptors) of the features, and the statistical parameter (average, median, variance, skewness, kurtosis) used in the image features (quantifying the features which were in the form of images), are given in the Supplementary Table A.1.

### Classifiers

Following feature selection, seven different classifiers, including cosine K-nearest neighbors (cosine KNN), fine KNN, subspace KNN, cross-entropy decision trees, RUSBoosted trees, cubic support vector machine (cubic SVM), and random forest were used for classification, and they were repeated across 100 repetitions of 10-fold cross-validation using bootstrapped subsets. In addition, the reported results are the average of these 100 repetitions. In other words, at each iteration of classification, training, and validation set were randomly selected, and after 100 repetitions of classification, the results of 100 times of classifications were averaged (Tables 5 and 6). This might help to evaluate the performance of a classifier under different conditions, such as variations in the training data or the classifier’s hyperparameters. Classifiers based on the top seven discriminating features were optimized on the training set. In the validation set, the class labels of EFs were predicted using these classifiers. The training set consisted of 46 patients, while the validation set consisted of 6 patients. Every time the validation set is selected, the subjects are collected in such a way to have an equal number of patients from all three classes (stratified with respect to the class). In other words, there are 2 subjects from each class in the validation set.

## Results

### Feature Analysis

Features selected by each feature selection algorithm are listed in Table 3. For example, Gabor_Median_W5O135 (WL = 5.66, orientation = 135) and Gabor_Average_W45O45 (WL = 45.25, orientation = 45) were selected by more than one feature selection algorithm. Among the algorithms, NCA performed better based on comparing classification results. Table 4 shows the details of the seven best-selected features by NCA. Features were chosen from different types of features, families, and groups. The median of Gabor with wavelength = 5.66 and orientation = 135 degrees, and an average of Gabor with wavelength = 45.25 and orientation = 45 degrees, both performed on the 2D image, were the most predictive features. The last two columns list the feature importance values and the *p* values between pairs of classes discriminated by each feature.

**Table 3:** The seven most predictive features in three feature selection methods. Again, bold features were shown as similar features in different feature selection methods

NCA	LASSO	MRMR
**Gabor_Median_W5O135**	Gabor_Average_W11O157	Haralick_Kurtosis_DiffAvg
**Gabor_Average_W45O45**	**Gabor_Median_W5O135**	LAWS_Kurtosis_W5W5
FO_Variance_ImgMed	LAWS_Skewness_R5E5	Gabor_Skewness_W8O45
NGTDM_Complexity	Gabor_Average_W45O112	Gabor_Kurtosis_W2O0
FO_Kurtosis_IntHist	**Gabor_Average_W45O45**	Gabor_Kurtosis_W5O22
GLCM_ClusterTendency	LAWS_Variance_L5S5	Gabor_Kurtosis_W2O67
Gabor_Kurtosis_W5O67	FO_Kurtosis_ImgMed	Gabor_Average_W5O112

**Table 4:** The seven best predictive features based on NCA feature selection. The last column shows *p* values between pairs of three classes based on Wilcoxon rank-sum tests. C1C2: pairs of class1 and class2, C1C3: pairs of class1 and class3, C2C3: pairs of class2 and class3

Feature name	Feature type	Feature group	Descriptor	Statistical parameter	Feature importance value	*p* value
Gabor_Median_W5O135	2D	Gabor	Wavelength = 5.66 Orientation = 135	Median	1	C1C2: 0.10 C1C3: < 0.001 C2C3: 0.20
Gabor_Average_W45O45	2D	Gabor	Wavelength = 45.25 Orientation = 45	Average	0.949	C1C2: 0.009 C1C3: 0.04 C2C3: 0.30
FO_Variance_ImgMed	2D 3in3 mask	First-order Gray Level	Image median	Variance	0.846	C1C2: 0.30 C1C3: 0.07 C2C3: 0.03
NGTDM_Complexity	2D/SERA	NGTDM	Complexity	–	0.814	C1C2: 0.20 C1C3: 0.70 C2C3: 0.80
FO_Kurtosis_IntHist	SERA	First Order	Intensity Histogram	Kurtosis	0.736	C1C2: 0.03 C1C3: 0.40 C2C3: 0.40
GLCM_ClusterTendency	3D/SERA	GLCM	Cluster Tendency	–	0.697	C1C2: 0.40 C1C3: 0.30 C2C3: 0.90
Gabor_Kurtosis_W5O67	2D 3in3 mask	Gabor	Wavelength = 5.66 Orientation = 67.5	Kurtosis	0.655	C1C2: 0.03 C1C3: 0.30 C2C3: 0.004

**Fig. 2:**
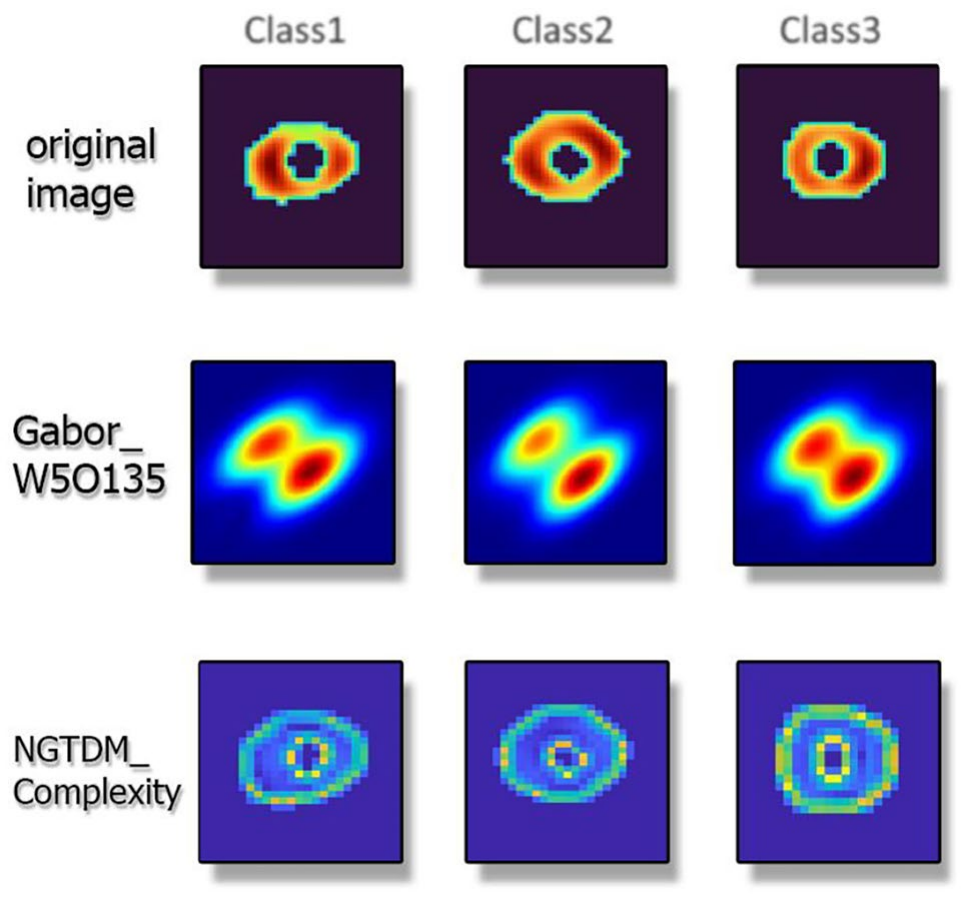
Display of two of the features selected by NCA, where each column represents a patient from classes 1 to 3. These two features include Gabor and NGTDM. It visually depicts the image-based features of patients belonging to three classes

Figure 2 presents a visualization of the scab and two of the top NCA selected features for three patients from different classes to showcase the textures of best predictive features in three different classes.

**Fig. 3:**
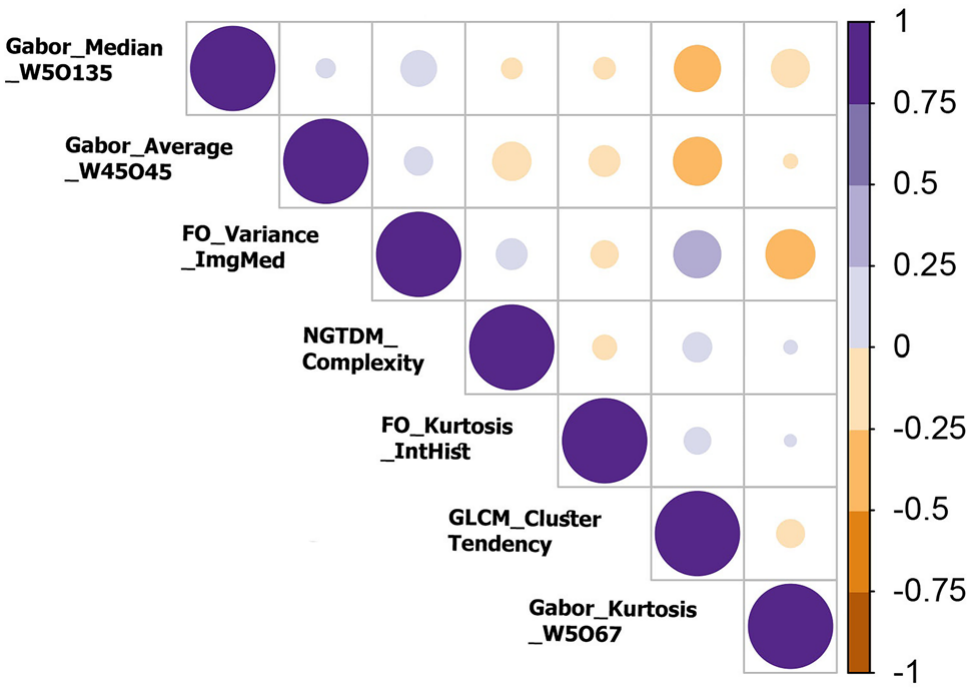
NCA selected features’ correlation analysis. Smaller and brighter circles illustrate lower correlation values than larger and darker ones used for higher correlations. It indicates a low correlation for most of the features

Correlation between NCA selected features was calculated, and the results can be seen in Fig. 3. Lower and higher correlations are indicated by smaller/lighter and bigger/darker circles, respectively. Highest correlations were between GLCM_ClusterTendency and Gabor_Median_W5O135, Gabor_Average_W45O45 and GLCM_ClusterTendency, FO_Variance_ImgMed and GLCM_ClusterTendency, and also Gabor_Kurtosis_W5O67 and FO_Variance_ImgMed.

**Fig. 4:**
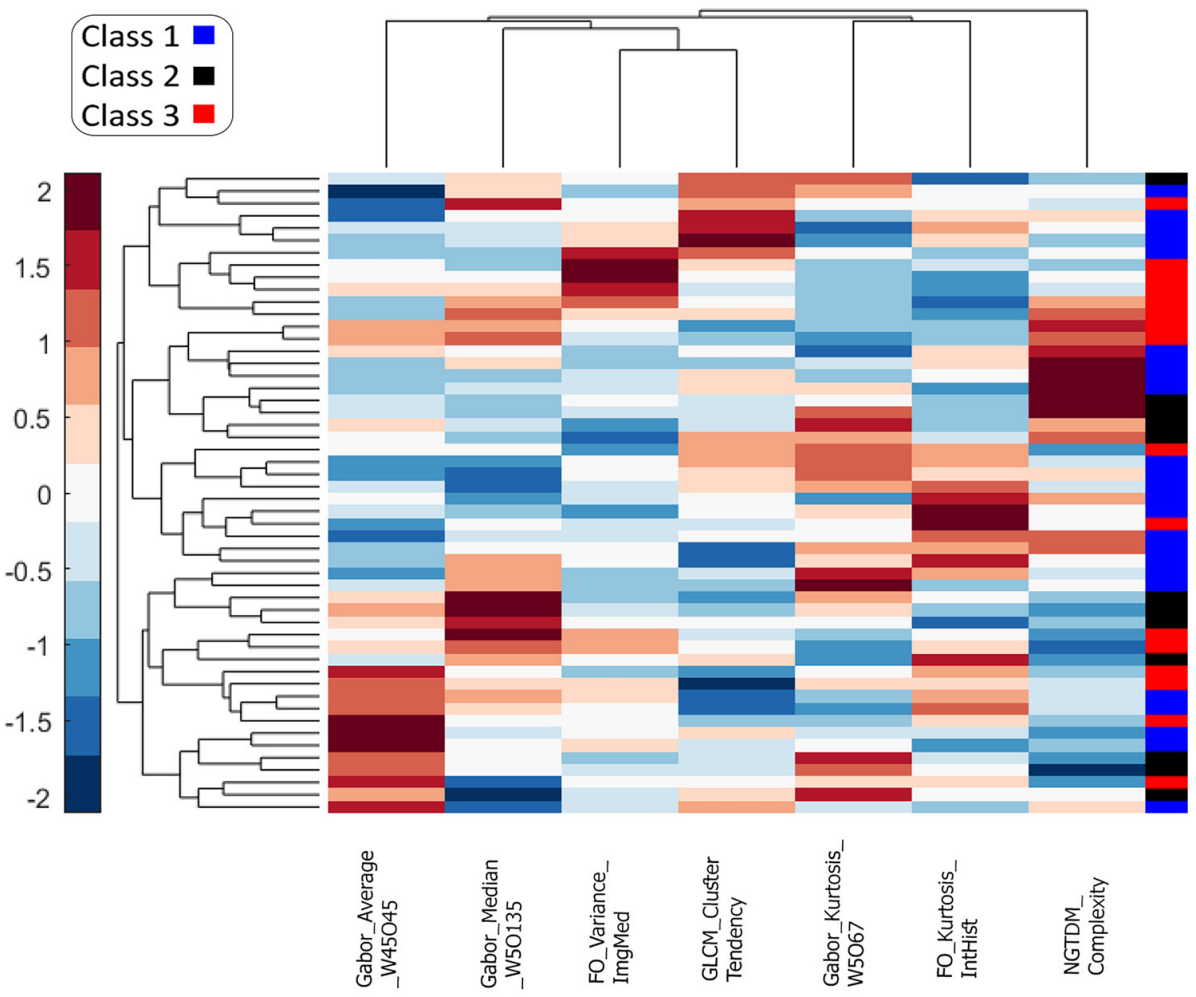
The seven selected features and their classes are depicted. Each row displays the feature normalized values for each patient, suggesting that values of features from specific classes tend to have similar values, while values of features from different classes tend to have roughly distinguishable values, highlighting the fact that classes are not properly classified

As shown in Fig. 4, the most discriminating features have been used in the feature expression-based cluster gram (hierarchical clustering). A number of the texture features show differential expression between the three classes. Based on the values of seven features, hierarchical clustering roughly divides values of the same classes close to each other. According to the three-color bar on the right, the classes are well separated in parts, but this method has not generally achieved complete separation.

**Fig. 5:**
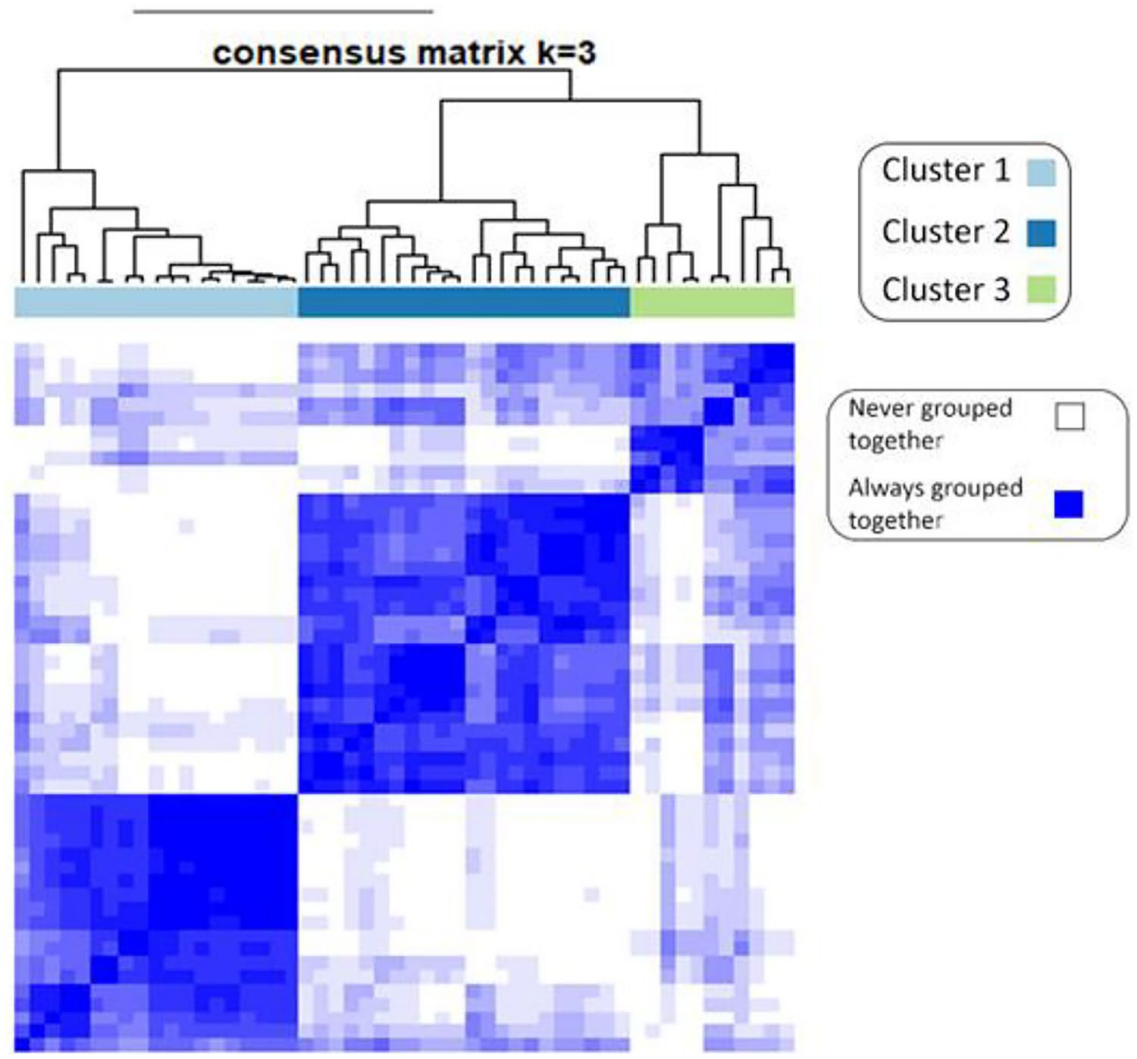
Based on a combination of top features, consensus clustering is shown. This graph shows three grouped clusters without labels and clearly shows three distinct clusters of features

Using the ConsensusClusterPlus package [33] in R [34], consensus clustering [35] was performed to determine instinctively how the patients were divided into three clusters (classes) based on seven selected features. Clustering three classes into three clusters of consensus resulted in 26% of class 1 for cluster 1, 63% of class 2 for cluster 2, and 54% of class 3 for cluster 3. To be more precise, it shows that 26% of class 1 patients are in cluster 1 of consensus clustering, 63% of class 2 patients are in cluster 2, and 54% of class 3 patients are in cluster 3. A measure of similarity between the three classes was determined based on the distance between the top seven features. The hierarchical consensus clustering (*k* = 3) for one thousand iterations with Pearson distance was performed on 80 percent (each iteration) of the random data sets. Figure 5 shows three grouped clusters without inherently considering labels.

### Model Analysis

Table 5 represents the performance of all models (combinations of seven classifiers and three feature selection algorithms) using AUC, sensitivity, specificity, precision, and F-score parameters while reporting their average for the classification of three classes. The classifiers were trained with 100 repetitions of 10-fold cross-validation using bootstrapped subsets, and the average values of the above parameters in 100 iterations are listed in Table 5. The best performances were achieved from models using NCA features in general, and the highest model was a fine KNN classifier trained on NCA features, achieving an average accuracy, AUC, sensitivity, specificity, precision, and F-score of 0.84, 0.83, 0.75, 0.87, 0.78, and 0.76, respectively.

**Table 5:** The average values of 100 times classifying for testing data are shown. Results include the mean value of accuracy, AUC, sensitivity, specificity, precision, and F-score for three classes. Feature selection methods are shown in the first column, including NCA, MRMR, and LASSO. The best values of each evaluation parameter are bolded

	Classifiers	Accuracy	AUC	Sensitivity	Specificity	Precision	F-score
NCA	Cosine KNN	**0.82**	**0.87**	**0.72**	**0.85**	**0.74**	**0.72**
	Fine KNN	**0.84**	**0.83**	**0.75**	**0.87**	**0.78**	**0.76**
	Subspace KNN	**0.80**	**0.84**	**0.69**	**0.84**	**0.74**	**0.69**
	Cross-entropy decision tree	0.76	0.75	0.61	0.82	0.61	0.60
	RUSBoosted trees	0.78	0.76	0.66	0.84	0.66	0.65
	Cubic SVM	0.75	0.80	0.62	0.81	0.65	0.60
	Random forest	**0.79**	**0.88**	**0.64**	**0.83**	**0.67**	**0.65**
MRMR	Cosine KNN	0.60	0.59	0.38	0.69	0.35	0.37
	Fine KNN	0.61	0.57	0.40	0.70	0.39	0.39
	Subspace KNN	0.61	0.56	0.39	0.68	0.37	0.37
	Cross-entropy decision tree	0.64	0.68	0.44	0.72	0.45	0.44
	RUSBoosted trees	0.65	0.62	0.48	0.74	0.47	0.46
	Cubic SVM	0.63	0.57	0.45	0.72	0.42	0.42
	Random forest	0.68	0.71	0.47	0.74	0.48	0.47
LASSO	Cosine KNN	0.72	0.81	0.54	0.78	0.55	0.53
	Fine KNN	0.75	0.69	0.58	0.80	0.60	0.59
	Subspace KNN	0.75	0.74	0.57	0.79	0.61	0.58
	Cross-entropy decision tree	0.75	0.76	0.63	0.81	0.63	0.62
	RUSBoosted trees	0.77	0.80	0.65	0.83	0.65	0.64
	Cubic SVM	0.73	0.74	0.56	0.79	0.57	0.55
	Random forest	0.76	0.81	0.59	0.81	0.60	0.58

**Fig. 6:**
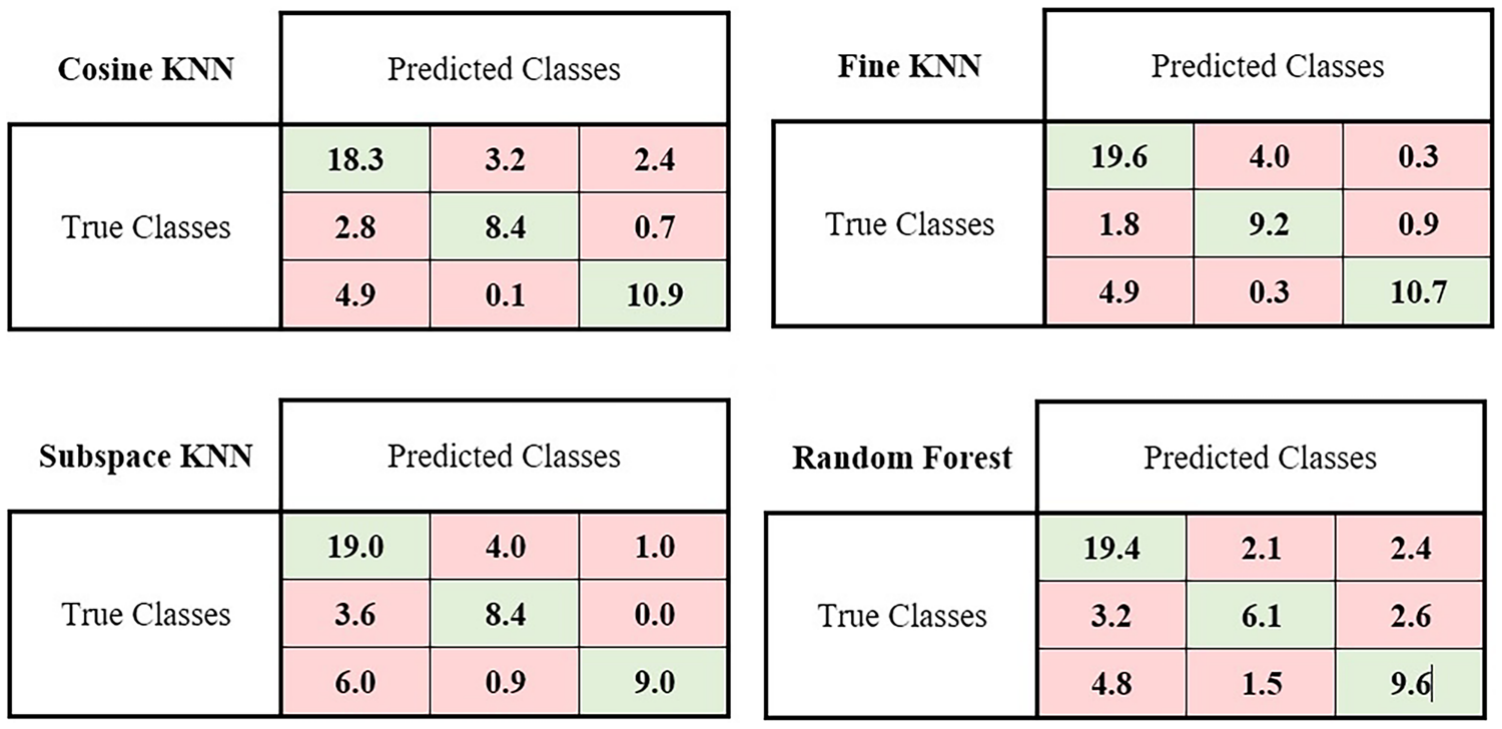
Confusion matrix of the four best models depicting the mean of 100 times classification for each of the values

Table 6 lists the details (mean ± standard deviation) of the results of the top 4 models (all trained on selected features by NCA), including accuracy, AUC, sensitivity, specificity, precision, and F-score for each class, separately. Bold values are displayed for the highest performance for each class. For example, fine KNN had the best performance for classifying classes one and three, and cosine KNN for classifying the second class. The confusion matrix for the four best models, namely, fine KNN and cosine KNN, can be found in Fig. 6. Values are presented for the average of 100 iterations of classification and are shown in two decimal order.

**Table 6:** The four best predictive models, all chosen by NCA feature selection. Accuracy, AUC, sensitivity, specificity, precision, and F-score are shown separately for each class. The best classifiers were the cosine KNN, fine KNN, subspace KNN, and random forest. The best performances for each class are shown in bold

	Classifiers	Classes	Accuracy (mean ± SD)	AUC (mean ± SD)	Sensitivity (mean ± SD)	Specificity (mean ± SD)	Precision (mean ± SD)	F-score (mean ± SD)
**NCA**	Cosine KNN	Class 1	0.74 ± 0.033	0.83 ± 0.083	0.76 ± 0.047	0.72 ± 0.041	0.71 ± 0.035	0.73 ± 0.036
		Class 2	**0.87 ± 0.023**	**0.92 ± 0.092**	**0.70 ± 0.067**	**0.92 ± 0.018**	**0.72 ± 0.052**	**0.71 ± 0.053**
		Class 3	0.84 ± 0.024	0.85 ± 0.085	0.69 ± 0.045	0.91 ± 0.028	0.78 ± 0.055	0.73 ± 0.039
	Fine KNN	Class 1	**0.79 ± 0.029**	**0.81 ± 0.080**	**0.82 ± 0.045**	**0.76 ± 0.039**	**0.75 ± 0.032**	**0.78 ± 0.031**
		Class 2	0.86 ± 0.022	0.84 ± 0.084	0.77 ± 0.051	0.89 ± 0.027	0.68 ± 0.052	0.72 ± 0.038
		Class 3	**0.87 ± 0.021**	**0.83 ± 0.083**	**0.67 ± 0.052**	**0.97 ± 0.020**	**0.90 ± 0.054**	**0.77 ± 0.044**
	Subspace KNN	Class 1	0.72 ± 0.034	0.82 ± 0.081	0.79 ± 0.043	0.66 ± 0.053	0.67 ± 0.036	0.72 ± 0.032
		Class 2	0.84 ± 0.022	0.88 ± 0.088	0.70 ± 0.064	0.88 ± 0.020	0.63 ± 0.046	0.66 ± 0.047
		Class 3	0.85 ± 0.026	0.83 ± 0.083	0.57 ± 0.073	0.97 ± 0.025	0.91 ± 0.078	0.69 ± 0.060
	Random forest	Class 1	0.76 ± 0.037	0.89 ± 0.089	0.81 ± 0.051	0.71 ± 0.059	0.71 ± 0.043	0.75 ± 0.035
		Class 2	0.82 ± 0.037	0.90 ± 0.090	0.51 ± 0.012	0.91 ± 0.032	0.63 ± 0.010	0.56 ± 0.010
		Class 3	0.78 ± 0.039	0.84 ± 0.084	0.60 ± 0.080	0.86 ± 0.046	0.66 ± 0.084	0.63 ± 0.066

**Fig. 7:**
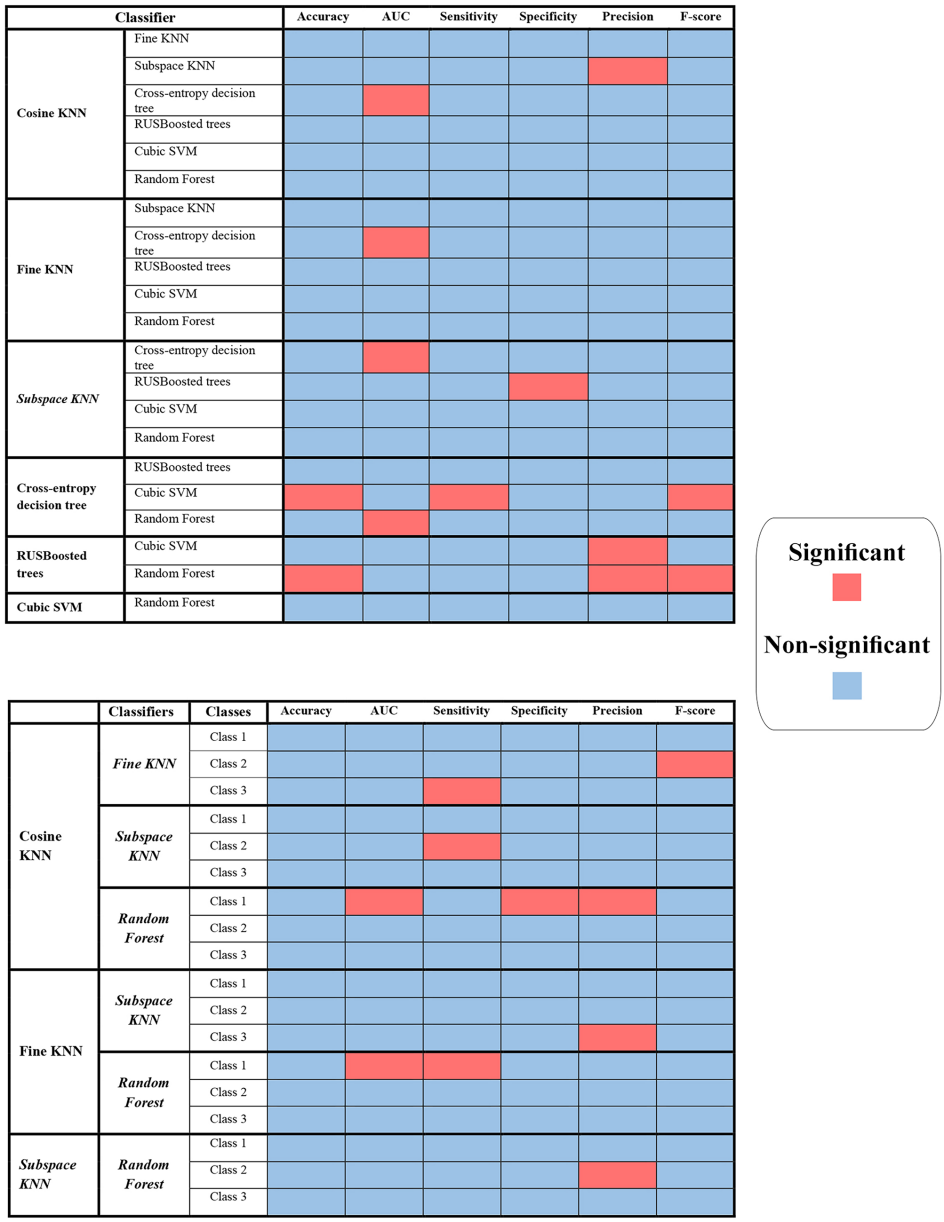
Comparison of the performance of seven classifiers based on the NCA feature selection method using the Wilcoxon rank-sum test. The top panel illustrates the p-values obtained from comparing the evaluation metrics of seven classifiers, including accuracy, AUC, sensitivity, specificity, precision, and F-score. The lower panel displays the *p* values obtained from comparing the top four classifiers based on their evaluation metrics, compared on a per-class basis

Figure 7 presents the *p* values obtained from the Wilcoxon rank-sum test for comparing the performance of classifiers based on the NCA feature selection method. The threshold for statistical significance was set to 0.05. The upper panel of Fig. 7 compares the performances of seven classifiers, indicating that most of them exhibit significant differences in their classification performance (Table 5). Therefore, the models differ significantly from each other, with some outperforming others. The lower panel in Fig. 7 displays the *p* values obtained from comparing the top four classifiers based on their evaluation metrics (Table 6). The metrics are compared per class and the p-values for each metric are displayed separately, indicating the significance of the difference in classifier performance. This graph also confirms that the models have significantly different results from each other, with some exhibiting significantly higher performance.

**Fig. 8:**
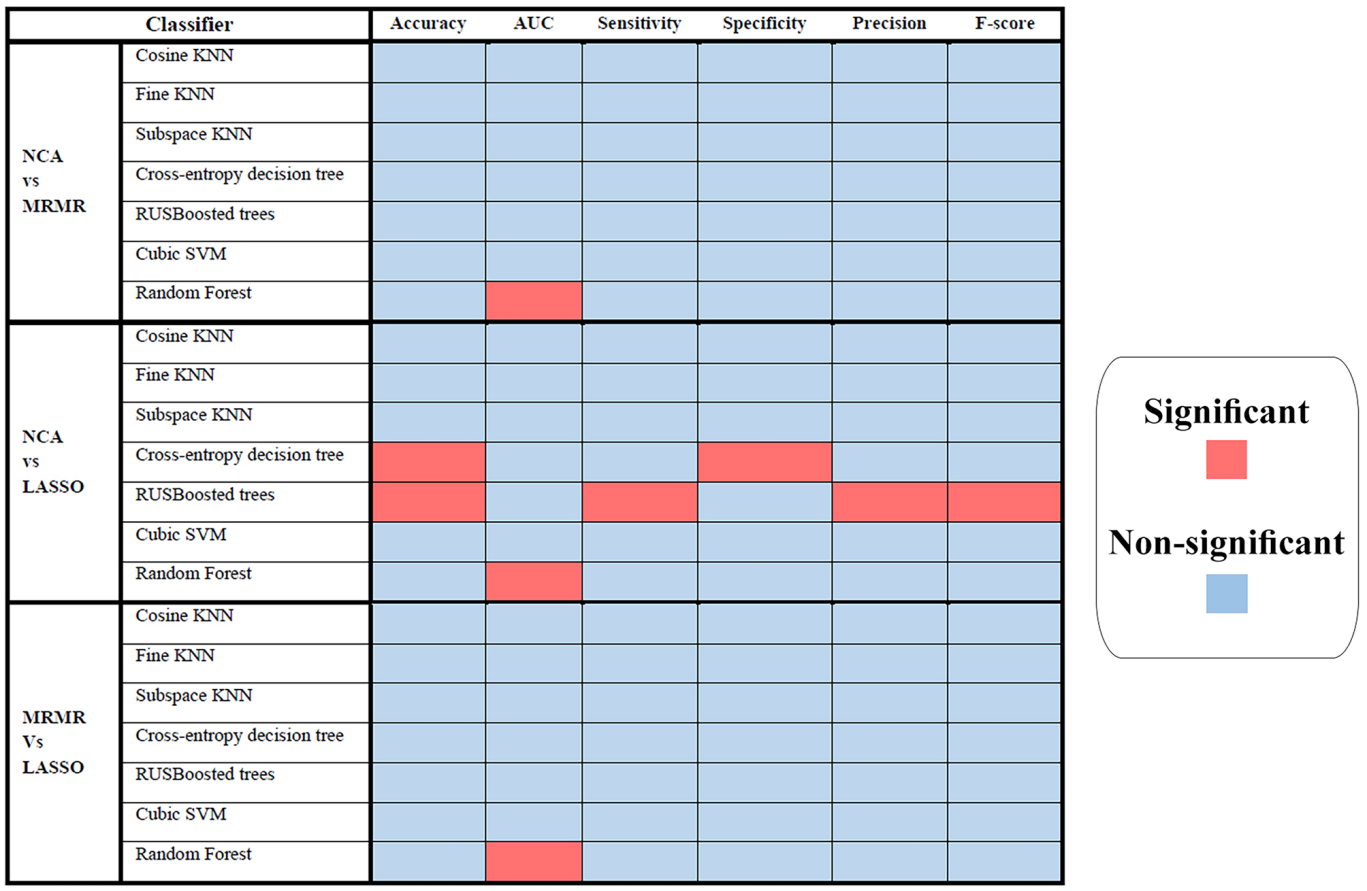
*p* Values obtained from the Wilcoxon rank-sum test comparing the performance of three feature selection methods, namely NCA, MRMR, and LASSO in seven different classifiers with evaluation metrics including accuracy, AUC, sensitivity, specificity, precision, and F-score

Figure 8 depicts the *p* values derived from the Wilcoxon rank-sum test utilized to evaluate the performance of classifiers based on three distinct feature selection techniques: NCA, MRMR, and LASSO. The statistical significance threshold is set at 0.05. The comparative analysis of the performance of the three feature selection methods on seven classifiers demonstrates that most of the classifiers exhibited significant differences in their classification performance. Consequently, based on the evaluation metrics, it can be concluded that the models differ significantly depending on the feature selection methods employed. Notably, the only exception to this finding is the RUSBoosted trees classifier, which revealed high *p* values when comparing the NCA and LASSO methods, implying no significant differences between the two techniques. However, in all other cases, there is a significant difference between the performance of the classifiers utilizing different feature selection methods.

**Fig. 9:**
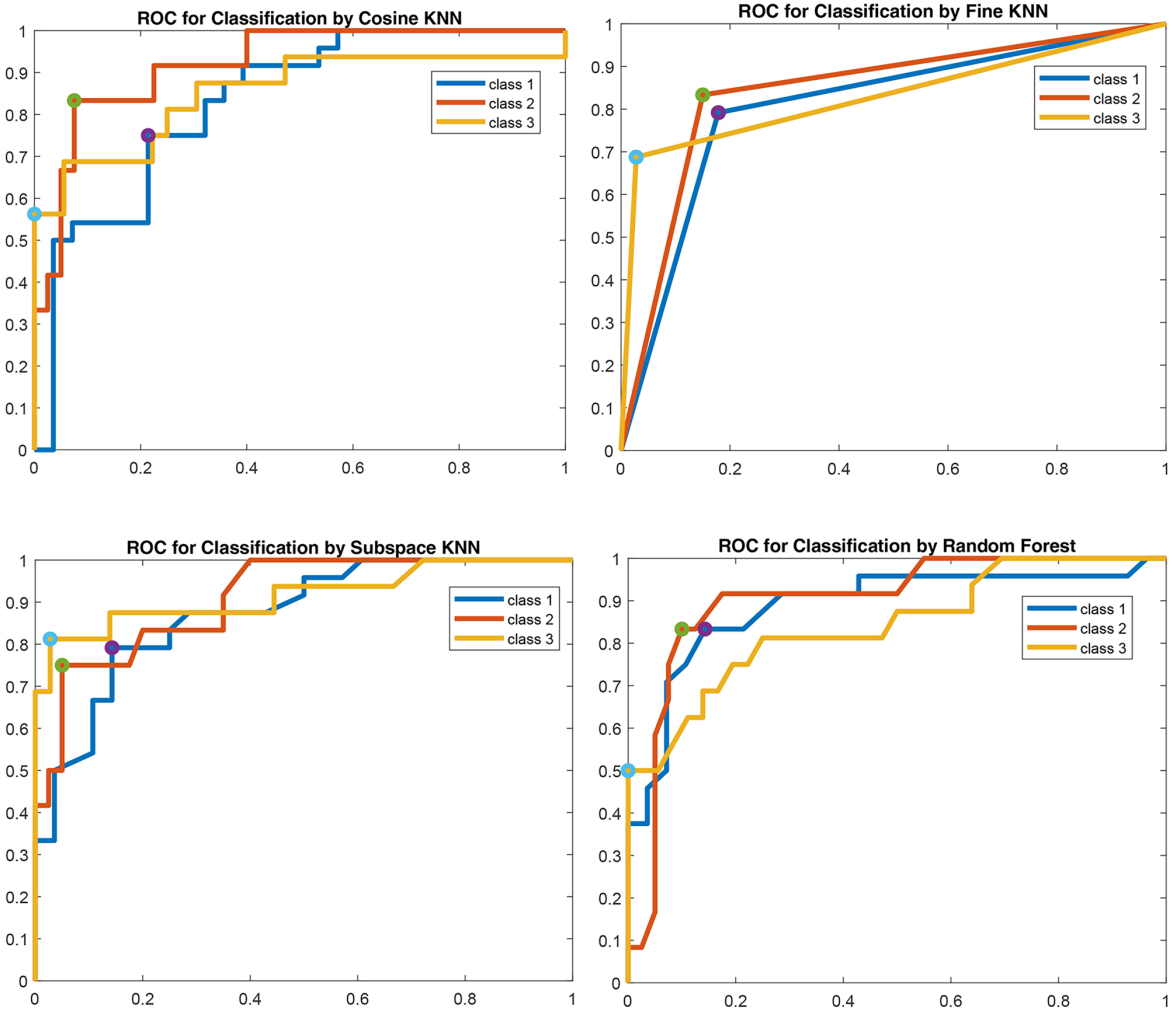
ROC of cosine KNN, fine KNN, subspace KNN, and random forest classifiers for three classes. The AUC of the classifiers is greater than 0.5 for all three classes. In cosine KNN, the optimal point for class 1, class 2, and class 3, respectively, was 0.73, 0.83, and 0.58. Fine KNN’s optimal point was 0.78 for class 1, 0.82 for class 2, and 0.69 for class 3. In subspace KNN, the optimal point was 0.78 for class 1, 0.75 for class 2, and 0.81 for class 3. In random forest, the optimal point was 0.82 for class 1, 0.83 for class 2, and 0.50 for class 3. The x-axis represents the False Positive Rate (FPR) (1-specificity) and the y-axis represents the True Positive Rate (TPR) (sensitivity)

Figure 9 shows the ROC curve of cosine KNN, fine KNN, subspace KNN, and random forest classifiers. It shows one-time training results of all 100 times trained classifiers. The AUC results of these four classifiers and other classifiers are depicted in Tables 5 and 6. For example, cosine KNN results demonstrated 0.83 AUC for class 1, 0.92 AUC for class 2, and 0.85 AUC for class 3, and fine KNN results show 0.81 AUC for class 1, 0.84 AUC for class 2 and 0.83 AUC for class 3. Also, subspace KNN results were 0.82 AUC for class 1, 0.88 AUC for class 2, and 0.83 AUC for class 3, and random forest obtained 0.89 AUC for class 1, 0.90 AUC for class 2, and 0.84 AUC for class 3

## Discussion

In this study, we aimed to predict the revascularization outcome by post-PCI EF. Although machine learning approaches have already been used in MPI-SPECT, it has not been used to predict EF improvements after PCI. Previous studies focused on clinical parameters to evaluate EF variations and efficacy factors on MI. Our work was geared toward more radiographic parameters where EF improvement is classified based on radiomic features. Physicians can benefit from the assessment proposed in this study and each patient’s clinical condition to determine whether PCI should be performed. Our analysis revealed seven radiomic features with considerable significance. After feature selection, selected features consisted mainly of Gabor, first-ordered, GLCM, and NGTDM features. The Gabor features are the most frequently used, followed by the First-ordered feature group, GLCM, and NGTDM. NCA feature selection algorithm appeared in all four best models, which shows NCA's superior performance. LASSO was more successful than MRMR among the other two feature selection methods based on classification results.

Two of the Gabor features were common in the two feature selection methods, and 3 of the top 7 features included Gabor filters, indicating this feature family's importance. Gabor filters [36] show a specific frequency content in a certain direction and a localized region, where the frequency in the image indicates the intensity variations. The first feature is the median of the Gabor image, and the second feature is the average of the Gabor image. The intensity of the image shows myocardial perfusion, and its changes show the difference in perfusion in different areas of the myocardium. Thus, the median and average of the frequency content (Gabor image) of the MPI-SPECT show the differences in perfusion in specific areas and directions of the myocardium. These differences in perfusion can be due to scattered and heterogenous regions of myocardial hibernation, infarction, scar, etc., which respond differently to revascularization. Oxygen and nutrition supplied by the revascularization may revive the hibernated regions on the myocardium leading to an increase in EF, which is not the case for severely infarcted zones [25]. The third feature contains the median of the 3 × 3 masks of the image, which moves along the entire image and finally presents an image where each pixel is the median of the 3 × 3 mask. In the end, the variance of the median image was calculated. The median shows the intensity value in the middle of the mask's intensity range, and the variance of the median image indicates how much the intensity values for each mask vary from the median value across all masks used in the analysis. As a result, this feature somehow aligns with the hypothesis of the first and second features, which hypothesized the perfusion heterogeneity in the myocardium, representative of zones with different infarction levels, predicting the improvement of EF after revascularization. Similarly, the fourth discriminative feature, NGTDM complexity, supports our hypothesis by showing non-uniform and rapid changes in grey levels.

Based on a comparison of seven machine-learning classifiers in Table 5, fine KNN proved to be the most predictive with accuracy = 0.84, sensitivity = 0.75, specificity = 0.87, precision = 0.78, and F-score = 0.76. In addition, random forest with AUC = 0.88 showed better results according to AUC values. Based on unbalanced data between classes, specificity, and sensitivity are more appropriate evaluation metrics. Regarding sensitivity and specificity, fine KNN with sensitivity = 0.75 and specificity = 0.87, which are acceptable values, also performed well. Radiomic features can be affected by the variability of features due to different scanners, acquisition protocols, segmentations, and processing protocols in MPI-SPECT [37, 38]. In this study, a unique gamma camera was used; thus, possible variations were offset; however, to get more reproducible results, vast and heterogenous datasets from multiple centers are needed. Participants underwent revascularization and MPI-SPECT using a specific gamma camera and a prior- and post-PCI echocardiogram as part of the study. However, due to the difficult nature of the data acquisition for this study, the number of patients was limited. The number of patients for each class was 24 for class 1, 12 for class 2, and 16 for class 3. Hence, a slight imbalance existed in the distribution of classes in our dataset. Wilcoxon rank-sum between classes was used in this regard, which indicates that five of the predictive features had a significant difference for at least one pair of classes. An analysis of the selected radiomic features using correlation was illustrated in Fig. 3. Low correlation (independent information) is seen in most features. The discriminability of the identified features was also illustrated using clustering methods.

Prediction of early coronary revascularization (ECR) by MPI-SPECT has been studied previously [39–41]. In [40], clinical characteristics like hypertension, dyslipidemia, smoking, family history, stress testing, and interpretation of MPI-SPECT by nuclear cardiologist experts have been used as predictors in machine learning algorithms. Then physician’s assessments were compared with machine learning assessments. The ability of ML to predict early revascularization in patients with suspected CAD was superior to the ability of individual quantitative measures, such as stress TPD, combined-view stress TPD, and ischaemic TPD, for each vessel and patient. Furthermore, ML was more accurate than the clinical interpretation by a human expert for each patient. Arsanjani et al. [21] examined the effectiveness of integrating clinical data with quantitative image features derived from MPI-SPECT to predict early revascularization in patients with suspected CAD using LogitBoost. In [42], robustness, repeatability, and reproducibility of cardiac SPECT radiomic features have been investigated as a phantom study. They examined the reproducibility of cardiac SPECT radiomic features under different imaging settings, which included reconstructing algorithms, the number of iterations and subsets, matrix size, attenuation correction, number of views, and post-reconstruction filters. In [25], radiomics were used to predict myocardial function improvement after CABG surgery in cardiac MR images, and the SCAD-penalized SVM approach obtained an AUC of 0.784. Hajianfar et al. [43] used MPI-SPECT with different reconstruction parameters to study the impact of ComBat harmonization on radiomics features. According to the Kruskal-Wallis test, 11, 10, 0, 21, and 1 features before ComBat harmonization had significant differences over the reconstruction method, filter, order, cutoff, and iteration subset, respectively. After ComBat harmonization, all features had no significant differences. As a result of applying ComBat harmonization, it could provide a solution to this problem, improving the reproducibility of radiomics features derived from different reconstruction methods.

Consensus clustering was performed to identify the stability and discrimination power of the predicted classes on the data set. Based on a consensus clustering approach, we investigated the correlation within and between classes for the features that were distinguished during feature selection (Fig. 5). There was a higher probability of belonging to a cluster when two radiomic features were close to each other. The basic idea is to perform clustering on the same dataset multiple times with random initialization and selection of clustering algorithms to minimize the risk of overfitting and instability of the clustering results. Features belonging to a cluster should have a high intra-class correlation (ICC), whereas features belonging to different clusters should have a lower correlation. As shown in Fig. 5, during 1000 iterations, the more pairs of patients are placed together, the bluer the color becomes, and the fewer pairs of patients are placed, the whiter the color becomes, and finally, the shading takes place between the two. In other words, it shows how often a pair of patients were paired up. A confusion matrix for four of the best models is shown in Fig. 6, with the table values representing the average of 100 classifications. Cosine KNN correctly predicted 18.3 of 24 patients in class 1, while fine KNN, subspace KNN, and random forest correctly diagnosed 19.6, 19.0, and 19.4, respectively. Also, in class 2, out of 12 patients, 8.4, 9.2, 8.4, and 6.1 were correctly diagnosed in cosine KNN, fine KNN, subspace KNN, and random forest, respectively, and in class 3, among 16 patients, 10.9, 10.7, 9.0, and 9.6 were correctly diagnosed in cosine KNN, fine KNN, subspace KNN, and random forest, respectively. It has been found that the models have demonstrated fair values for the classification of three classes. A limitation of the study is the relatively small number of patients due to the difficult follow-up nature of the study. The study also had the limitation of collecting data from a single site, which may undermine the study’s robustness. An investigation of the relationship between features and EF improvement following revascularization could be conducted with a larger dataset. In addition, repeatability (specific scanner/protocol) and reproducibility (different scanner/protocol) of radiomics and machine learning for post-revascularization EF improvement in the same patients have not been evaluated and can be investigated in future studies. Also, an analysis could be attained using the 17-segment model of the revascularized artery reported on a PCI report. Moreover, further work could be conducted using deep learning methods with a larger sample size.

## Conclusion

In this study, we employed a combination of 2D and 3D radiomics approaches for EF improvement prediction after revascularization with respect to three classes of classification. It was shown that radiomic features are related to post-revascularization EF improvement, and it can predict EF improvement with insignificant error with convenient accuracy. Potentially, these findings can have a significant clinical impact on decision-making. For example, it can help physicians assess the cost and benefits of PCI procedures in patients with particular risk factors. Furthermore, it can help by reducing the risk of infection and complications, reducing the cost and duration of hospitalization [41–43].

## Supplementary Information

Below is the link to the electronic supplementary material.

Supplementary file1 (DOCX 16.2 KB)

## Data Availability

Not applicable.

## Abbreviations

CAD: Coronary artery disease
LV: Left ventricle
MI: Myocardial infarction
PCI: Percutaneous coronary intervention
CABG: Coronary artery bypass graft
EF: Ejection fraction
CAG: Coronary angiography
MPI-SPECT: Myocardial perfusion imaging with single-photon emission computed tomography
Cine-CMR: Cine cardiac magnetic resonance
LGE-CMR: Late gadolinium enhancement on cardiac MR
gSPECT: Electrocardiography-gated SPECT
FBP: Filtered back projection
ROI: Regions of interest
SERA: Standardized environment for radiomics analysis
IBSI: Image biomarker standardization initiative
GLCM: Gray level co-occurrence matrix
NCA: Neighborhood component analysis
MRMR: Minimum redundancy maximum relevance
LASSO: Least absolute shrinkage and selection operator
Cosine KNN: Cosine K-nearest neighbors
Cubic SVM: Cubic support vector machine
ECR: Early coronary revascularization
ICC: Intra-class correlation

## Code Availability

This study uses an open-source library including R and SERA software.
