# Supplementary material for: Post-revascularization Ejection Fraction Prediction for Patients Undergoing Percutaneous Coronary Intervention Based on Myocardial Perfusion SPECT Imaging Radiomics: a Preliminary Machine Learning Study
Source: J Digit Imaging. 2023 Apr 14;36(4):1348–63. doi: 10.1007/s10278-023-00820-1 (PMC10407007; doi:10.1007/s10278-023-00820-1)
Supplement: Supplementary file 1 — Supplementary file1 (DOCX 16.2 KB) [file 10278_2023_820_MOESM1_ESM.docx]

**Supplementary Table A.1.** It shows Features abbreviation with the details of the features parameters. It includes self-generated codes and SERA package.

| Feature Abbreviation | Feature type | Feature group | Descriptor | Statistical parameter |
| --- | --- | --- | --- | --- |
| Gabor_Median_W5O135 | 2D | Gabor | Wavelength=5.66  Orientation=135 | Median |
| Gabor_Average_W45O45 | 2D | Gabor | Wavelength=45.25  Orientation=45 | Average |
| FO_Variance_ImgMed | 2D 3in3 mask | First-order  Gray Level | Image Median | Variance |
| NGTDM_Complexity | 2D/SERA | NGTDM | Complexity | – |
| FO_Kurtosis_IntHist | SERA | First  Order | Intensity  Histogram | Kurtosis |
| GLCM_ClusterTendency | 3D/SERA | GLCM | Cluster  Tendency | – |
| Gabor_Kurtosis_W5O67 | 2D 3in3 mask | Gabor | Wavelength=5.66  Orientation=67.5 | Kurtosis |
| Gabor_Average_W11O157 | 2D | Gabor | Wavelength=11.31  Orientation=157.5 | Average |
| LAWS_Skewness_R5E5 | 2D | LAWS | R5E5 | Skewness |
| Gabor_Average_W45O112 | 2D | Gabor | Wavelength=45.25  Orientation=112.5 | Average |
| LAWS_Variance_L5S5 | 2D | LAWS | L5S5 | Variance |
| FO_Kurtosis_ImgMed | 2D 3in3 mask | First-order  Gray Level | Image Median | Kurtosis |
| Haralick_Kurtosis_DiffAvg | 2D 3in3 mask | Haralick | Difference Average | Kurtosis |
| LAWS_ Kurtosis_W5W5 | 2D | LAWS | W5W5 | Kurtosis |
| Gabor_Skewness_W8O45 | 2D | Gabor | Wavelength=8.20  Orientation=45 | Skewness |
| Gabor_ Kurtosis_W2O0 | 2D 3in3 mask | Gabor | Wavelength=2.83  Orientation=0 | Kurtosis |
| Gabor_ Kurtosis_W5O22 | 2D 3in3 mask | Gabor | Wavelength=5.66  Orientation=22.5 | Kurtosis |
| Gabor_ Kurtosis_W2O67 | 2D 3in3 mask | Gabor | Wavelength=2.83  Orientation=67.5 | Kurtosis |
| Gabor_Average_W5O112 | 2D 3in3 mask | Gabor | Wavelength=5.66  Orientation=112.5 | Average |
